# Pyridinium *cis*-diaqua­bis­(oxalato-κ^2^ *O*,*O*′)chromate(III)

**DOI:** 10.1107/S1600536812044303

**Published:** 2012-10-31

**Authors:** Justin Nenwa, Olivier Befolo, Bebga Gouet, Mohammed Mbarki, Boniface P. T. Fokwa

**Affiliations:** a Department of Inorganic Chemistry, University of Yaounde I, POB 812 Yaounde, Cameroon; b Higher Teacher Training College, POB 47, University of Yaounde 1, Cameroon; c Institut für Anorganische Chemie, RWTH Aachen, D-52056 Aachen, Germany

## Abstract

The title compound, (C_5_H_6_N)[Cr(C_2_O_4_)_2_(H_2_O)_2_], contains one protonated pyridine mol­ecule and one [Cr(C_2_O_4_)_2_(H_2_O)_2_]^−^ complex anion in the asymmetric unit. The Cr^III^ in the complex anion is coordinated in a distorted octa­hedral environment by two O atoms from two *cis* water mol­ecules and four O atoms from two chelating oxalate dianions. The crystal packing is stabilized by inter­molecular N—H⋯O(oxalate) and O—H⋯O(oxalate) hydrogen bonds and by π–π stacking inter­actions (centroid–centroid distance = 3.602 Å) between pyridine rings, thereby building up a three-dimensional network.

## Related literature

For the structural characterization of organic–inorganic salts containing the [Cr(C_2_O_4_)_2_(H_2_O)_2_]^−^ anion, see: Bélombé *et al.* (2009 ▶); Nenwa *et al.* (2010 ▶, 2012 ▶); Chérif *et al.* (2011 ▶); Chérif, Abdelhak *et al.* (2012 ▶); Chérif, Zid *et al.* (2012 ▶).
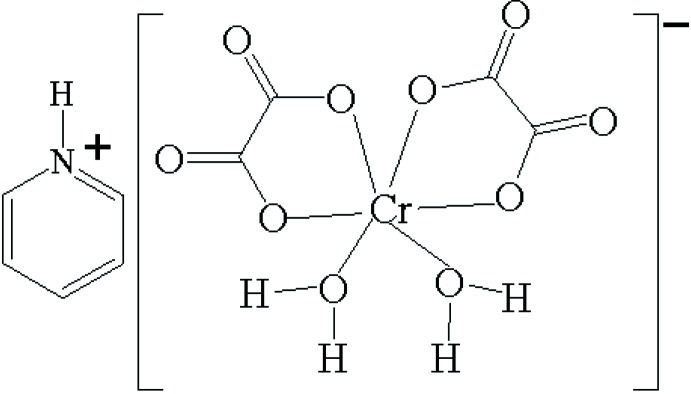


## Experimental

### 

#### Crystal data

(C_5_H_6_N)[Cr(C_2_O_4_)_2_(H_2_O)_2_]
*M* *_r_* = 344.18
Monoclinic, 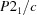

*a* = 7.479 (2) Å
*b* = 24.700 (8) Å
*c* = 7.056 (2) Å
β = 107.744 (6)°
*V* = 1241.4 (6) Å^3^
*Z* = 4
Mo *K*α radiation
μ = 0.98 mm^−1^
*T* = 100 K
0.15 × 0.04 × 0.04 mm

#### Data collection

Bruker SMART APEX CCD diffractometer
Absorption correction: multi-scan (*SADABS*; Bruker, 2004 ▶) *T*_min_ = 0.862, *T*_max_ = 0.961
18649 measured reflections
3703 independent reflections
2580 reflections with *I* > 2σ(*I*)
*R*_int_ = 0.106

#### Refinement

*R*[*F* ^2^ > 2σ(*F* ^2^)] = 0.061
*wR*(*F* ^2^) = 0.160
*S* = 1.13
3703 reflections
206 parameters
5 restraints
H atoms treated by a mixture of independent and constrained refinement
Δρ_max_ = 0.59 e Å^−3^
Δρ_min_ = −0.69 e Å^−3^

### 

Data collection: *SMART* (Bruker, 2004 ▶); cell refinement: *SAINT* (Bruker, 2004 ▶); data reduction: *SAINT*; program(s) used to solve structure: *SHELXS97* (Sheldrick, 2008 ▶); program(s) used to refine structure: *SHELXL97* (Sheldrick, 2008 ▶); molecular graphics: *DIAMOND* (Brandenburg, 2010 ▶); software used to prepare material for publication: *WinGX* (Farrugia, 1999 ▶).

## Supplementary Material

Crystal structure: contains datablock(s) I, global. DOI: 10.1107/S1600536812044303/lr2086sup1.cif

Structure factors: contains datablock(s) I. DOI: 10.1107/S1600536812044303/lr2086Isup2.hkl

Additional supplementary materials:  crystallographic information; 3D view; checkCIF report

## Figures and Tables

**Table 1:** Hydrogen-bond geometry (Å, °)

*D*—H⋯*A*	*D*—H	H⋯*A*	*D*⋯*A*	*D*—H⋯*A*
N1—H1⋯O24	0.86	2.10	2.783 (4)	136
N1—H1⋯O23	0.86	2.17	2.901 (4)	143
O*W*1—H1*B*⋯O12^i^	0.81 (2)	1.94 (2)	2.720 (4)	162 (5)
O*W*1—H1*A*⋯O23^ii^	0.86 (5)	1.84 (5)	2.680 (4)	168 (5)
O*W*2—H2*A*⋯O13^iii^	0.81 (2)	1.98 (3)	2.732 (4)	154 (4)
O*W*2—H2*B*⋯O14^iv^	0.82 (2)	1.83 (2)	2.639 (4)	173 (5)

Symmetry codes: (i) 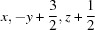; (ii) 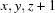; (iii) 
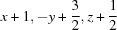
; (iv) 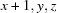.

**Fig. 1.**
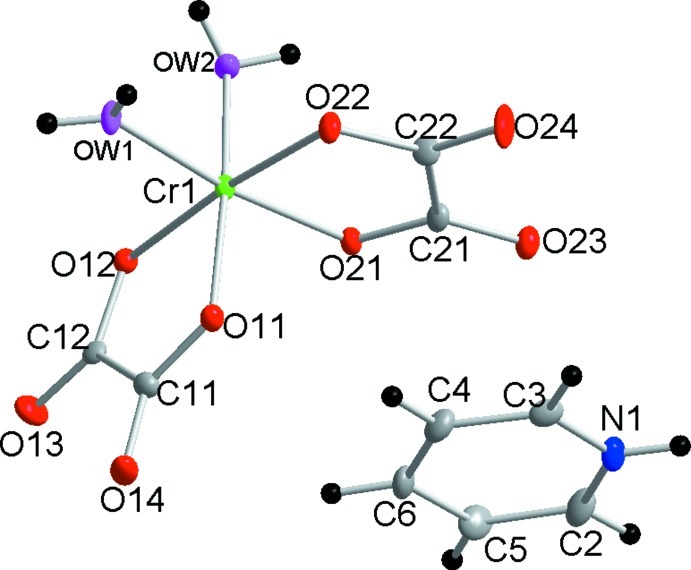
The asymmetric unit of the title compound with displacement ellipsoids drawn at the 50% probability level.

**Fig. 2.**
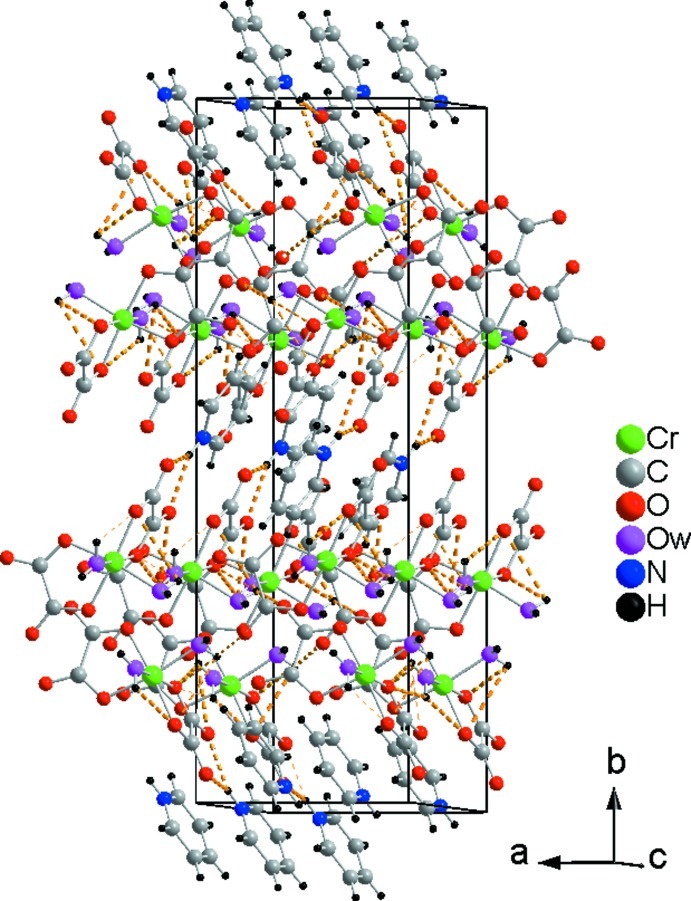
Crystal packing of the title compound, showing the components linked via N–H···O and O–H···O hydrogen bonds (dashed lines) forming a three-dimensional network. π–π stacking between the protonated pyridine rings is also observed.

## supplementary crystallographic information

### Comment

Recently, we have reported the structure of a few organic-inorganic hybrid salts involving various aromatic iminium cations and the complex anion, [Cr(C_2_O_4_)_2_(H_2_O)_2_]^-^ in *trans*-geometry (Bélombé *et al.*, 2009; Nenwa *et al.*, 2010, 2012). In the present investigation, we wish to report the structure of a homologous salt, containing the complex anion adopting the cis-geometry and protonated pyridinium as a counter cation. This *cis*-anion which is somewhat less common has been observed in a similar organic-inorganic hybrid salt, with 2-amino-5-chloropyridinium as the organic cation (Chérif, Abdelhak *et al.*, 2012).

The asymmetric unit of the title compound, (C_5_H_6_N)[Cr(C_2_O_4_)_2_(H_2_O)_2_] which crstallizes in space group *P2_1_/c*, is shown in Fig. 1. The Cr^III^ site in the complex anion has a distorted octahedral coordination environment build up by two O atoms (OW1, OW2) from two *cis* water molecules and four O atoms (O11, O12, O21, O22) from two chelating oxalate dianions. The main geometric parameters of the (C_5_H_6_N)^+^ cation are in agreement with those found in salts with similar cationic entities (Bélombé *et al.*, 2009; Nenwa *et al.*, 2010; Nenwa *et al.*, 2012; Chérif *et al.*, 2011; Chérif, Abdelhak *et al.*, 2012; Chérif, Zid *et al.*, 2012). The bond distances in the complex anion (Table 1) are comparable with those reported in the 2-amino-5-chloropyridinium compound (Chérif, Abdelhak *et al.*, 2012). The crystal packing is stabilized by intermolecular N—H···O (oxalate) and O—H···O (oxalate) hydrogen bonds and by π–π stacking interactions [centroid-centroid distance = 3.602 Å] between pyridine rings, thereby building up a three-dimensional network (Table 2, Fig. 2).

### Experimental

Pyridine (1 mmol, 79.1 mg) and oxalic acid (2 mmol, 260 mg) were added in successive small portions in an aqueous solution (50 ml) of CrCl_3_.6H_2_O (1 mmol, 266.5 mg). The mixture was stirred for 4 h continuously. The final blue-violet solution obtained was left at room temperature and violet crystals suitable for X-ray diffraction were obtained after a few days.

### Refinement

The H atoms were positioned geometrically, with C—H, N—H distances of 0.95 and 0.86 Å respectively, and constrained to ride on their parent atoms, with *U*_iso_(H) = 1.2U_eq_(C,N). The water H atoms were initially located in a difference Fourier map and refined with distance restraints of d(O–H1) = 0.83 (2) with all *U*_iso_(H) values refined.

### Crystal data

**Table:**

(C_5_H_6_N)[Cr(C_2_O_4_)_2_(H_2_O)_2_]	*F*(000) = 700
*M**_r_* = 344.18	*D*_x_ = 1.842 Mg m^−^^3^
Monoclinic, *P*2_1_/*c*	Mo *K*α radiation, λ = 0.71073 Å
Hall symbol: -P_2ybc	Cell parameters from 3703 reflections
*a* = 7.479 (2) Å	θ = 1.7–30.9°
*b* = 24.700 (8) Å	µ = 0.98 mm^−^^1^
*c* = 7.056 (2) Å	*T* = 100 K
β = 107.744 (6)°	Needle, violet
*V* = 1241.4 (6) Å^3^	0.15 × 0.04 × 0.04 mm
*Z* = 4	

### Data collection

**Table:**

Bruker SMART APEX CCD diffractometer	3703 independent reflections
Radiation source: fine-focus sealed tube	2580 reflections with *I* > 2σ(*I*)
Graphite monochromator	*R*_int_ = 0.106
φ and ω scans	θ_max_ = 30.9°, θ_min_ = 1.7°
Absorption correction: multi-scan (*SADABS*; Bruker, 2004)	*h* = −10→10
*T*_min_ = 0.862, *T*_max_ = 0.961	*k* = −34→33
18649 measured reflections	*l* = −9→10

### Refinement

**Table:**

Refinement on *F*^2^	Primary atom site location: structure-invariant direct methods
Least-squares matrix: full	Secondary atom site location: difference Fourier map
*R*[*F*^2^ > 2σ(*F*^2^)] = 0.061	Hydrogen site location: inferred from neighbouring sites
*wR*(*F*^2^) = 0.160	H atoms treated by a mixture of independent and constrained refinement
*S* = 1.13	*w* = 1/[σ^2^(*F*_o_^2^) + (0.0636*P*)^2^] where *P* = (*F*_o_^2^ + 2*F*_c_^2^)/3
3703 reflections	(Δ/σ)_max_ = 0.001
206 parameters	Δρ_max_ = 0.59 e Å^−^^3^
5 restraints	Δρ_min_ = −0.69 e Å^−^^3^

### Special details

**Table:**

Geometry. All s.u.'s (except the s.u. in the dihedral angle between two l.s. planes) are estimated using the full covariance matrix. The cell s.u.'s are taken into account individually in the estimation of s.u.'s in distances, angles and torsion angles; correlations between s.u.'s in cell parameters are only used when they are defined by crystal symmetry. An approximate (isotropic) treatment of cell s.u.'s is used for estimating s.u.'s involving l.s. planes.
Refinement. Refinement of F^2^ against ALL reflections. The weighted R-factor wR and goodness of fit S are based on F^2^, conventional R-factors R are based on F, with F set to zero for negative F^2^. The threshold expression of F^2^ > 2σ(F^2^) is used only for calculating R-factors(gt) etc. and is not relevant to the choice of reflections for refinement. R-factors based on F^2^ are statistically about twice as large as those based on F, and R- factors based on ALL data will be even larger.

### Fractional atomic coordinates and isotropic or equivalent isotropic displacement parameters (Å^2^)

**Table:**

	*x*	*y*	*z*	*U*_iso_*/*U*_eq_	
Cr1	0.00150 (8)	0.67285 (2)	0.06297 (8)	0.00840 (15)	
C11	−0.3839 (5)	0.67581 (15)	−0.0409 (5)	0.0119 (7)	
C12	−0.3217 (5)	0.73552 (14)	−0.0479 (5)	0.0099 (7)	
C21	0.0773 (5)	0.61425 (14)	−0.2335 (5)	0.0110 (7)	
C22	0.1456 (5)	0.57906 (15)	−0.0428 (5)	0.0120 (7)	
O11	−0.2502 (3)	0.64143 (10)	0.0020 (4)	0.0113 (5)	
O12	−0.1425 (3)	0.74032 (10)	−0.0051 (4)	0.0105 (5)	
O13	−0.4367 (4)	0.77229 (11)	−0.0905 (4)	0.0177 (6)	
O14	−0.5518 (4)	0.66425 (11)	−0.0766 (4)	0.0170 (6)	
O21	0.0065 (3)	0.65975 (10)	−0.2098 (4)	0.0113 (5)	
O22	0.1238 (4)	0.60185 (10)	0.1125 (4)	0.0118 (5)	
O23	0.0948 (4)	0.59673 (10)	−0.3912 (4)	0.0137 (5)	
O24	0.2113 (4)	0.53428 (11)	−0.0504 (4)	0.0186 (6)	
OW1	0.0262 (4)	0.67909 (11)	0.3493 (4)	0.0139 (5)	
OW2	0.2371 (4)	0.71532 (11)	0.1053 (4)	0.0139 (5)	
N1	0.2547 (4)	0.48994 (13)	−0.3963 (5)	0.0148 (6)	
H1	0.2081	0.5152	−0.3419	0.018*	
C2	0.3312 (6)	0.44697 (16)	−0.2871 (6)	0.0177 (8)	
H2	0.3320	0.4446	−0.1553	0.021*	
C3	0.2476 (5)	0.49522 (15)	−0.5870 (6)	0.0147 (7)	
H3	0.1927	0.5256	−0.6590	0.018*	
C4	0.3220 (6)	0.45543 (16)	−0.6751 (6)	0.0172 (8)	
H4	0.3164	0.4583	−0.8082	0.021*	
C5	0.4083 (5)	0.40652 (15)	−0.3686 (6)	0.0180 (8)	
H5	0.4618	0.3765	−0.2930	0.022*	
C6	0.4059 (5)	0.41073 (16)	−0.5641 (6)	0.0161 (8)	
H6	0.4599	0.3839	−0.6212	0.019*	
H1B	−0.019 (6)	0.6988 (17)	0.415 (6)	0.032 (15)*	
H2A	0.310 (5)	0.721 (2)	0.214 (4)	0.033 (15)*	
H1A	0.051 (8)	0.6501 (17)	0.419 (9)	0.06 (2)*	
H2B	0.296 (6)	0.700 (2)	0.040 (6)	0.06 (2)*	

### Atomic displacement parameters (Å^2^)

**Table:**

	*U*^11^	*U*^22^	*U*^33^	*U*^12^	*U*^13^	*U*^23^
Cr1	0.0108 (3)	0.0067 (3)	0.0083 (3)	0.0012 (2)	0.0037 (2)	0.0006 (2)
C11	0.0116 (16)	0.0135 (17)	0.0112 (17)	0.0003 (13)	0.0043 (13)	0.0004 (14)
C12	0.0122 (16)	0.0114 (16)	0.0052 (16)	0.0005 (13)	0.0015 (13)	0.0007 (12)
C21	0.0141 (17)	0.0108 (17)	0.0102 (17)	−0.0011 (13)	0.0065 (14)	−0.0005 (13)
C22	0.0157 (17)	0.0120 (17)	0.0095 (17)	0.0011 (13)	0.0056 (14)	0.0046 (14)
O11	0.0123 (12)	0.0093 (12)	0.0129 (13)	−0.0006 (9)	0.0048 (10)	−0.0011 (9)
O12	0.0116 (12)	0.0086 (12)	0.0110 (13)	0.0004 (9)	0.0031 (10)	0.0003 (9)
O13	0.0148 (13)	0.0116 (13)	0.0250 (16)	0.0046 (10)	0.0035 (11)	0.0026 (11)
O14	0.0128 (12)	0.0175 (14)	0.0222 (15)	−0.0043 (10)	0.0073 (11)	−0.0070 (11)
O21	0.0132 (12)	0.0094 (12)	0.0123 (13)	0.0012 (9)	0.0053 (10)	−0.0014 (9)
O22	0.0187 (13)	0.0109 (13)	0.0074 (12)	0.0035 (10)	0.0061 (10)	0.0021 (10)
O23	0.0205 (13)	0.0112 (13)	0.0109 (13)	0.0017 (10)	0.0069 (10)	0.0004 (10)
O24	0.0351 (16)	0.0092 (13)	0.0144 (14)	0.0103 (11)	0.0119 (12)	0.0024 (10)
OW1	0.0217 (14)	0.0106 (13)	0.0106 (13)	0.0050 (11)	0.0069 (11)	−0.0003 (10)
OW2	0.0108 (12)	0.0163 (14)	0.0150 (14)	−0.0020 (10)	0.0044 (11)	−0.0048 (11)
N1	0.0198 (16)	0.0104 (15)	0.0167 (16)	−0.0004 (12)	0.0090 (13)	−0.0021 (12)
C2	0.028 (2)	0.0151 (19)	0.0117 (18)	−0.0035 (16)	0.0091 (16)	−0.0003 (14)
C3	0.0166 (18)	0.0114 (17)	0.0139 (18)	−0.0028 (13)	0.0013 (14)	0.0026 (14)
C4	0.024 (2)	0.0173 (19)	0.0100 (18)	−0.0015 (15)	0.0040 (15)	−0.0013 (14)
C5	0.024 (2)	0.0083 (17)	0.019 (2)	0.0024 (14)	0.0037 (16)	0.0042 (14)
C6	0.0199 (19)	0.0129 (18)	0.017 (2)	0.0007 (14)	0.0068 (15)	−0.0062 (14)

### Geometric parameters (Å, º)

**Table:**

Cr1—O11	1.959 (3)	OW1—H1B	0.814 (19)
Cr1—O22	1.960 (3)	OW1—H1A	0.86 (5)
Cr1—O12	1.963 (3)	OW2—H2A	0.806 (19)
Cr1—O21	1.963 (3)	OW2—H2B	0.818 (19)
Cr1—OW1	1.978 (3)	N1—C2	1.334 (5)
Cr1—OW2	1.993 (3)	N1—C3	1.337 (5)
C11—O14	1.237 (4)	N1—H1	0.8600
C11—O11	1.276 (4)	C2—C5	1.365 (5)
C11—C12	1.552 (5)	C2—H2	0.9300
C12—O13	1.224 (4)	C3—C4	1.369 (5)
C12—O12	1.286 (4)	C3—H3	0.9300
C21—O23	1.238 (4)	C4—C6	1.388 (5)
C21—O21	1.275 (4)	C4—H4	0.9300
C21—C22	1.552 (5)	C5—C6	1.378 (6)
C22—O24	1.218 (4)	C5—H5	0.9300
C22—O22	1.285 (4)	C6—H6	0.9300
			
O11—Cr1—O22	92.85 (11)	C12—O12—Cr1	115.7 (2)
O11—Cr1—O12	82.18 (10)	C21—O21—Cr1	114.0 (2)
O22—Cr1—O12	174.30 (11)	C22—O22—Cr1	114.4 (2)
O11—Cr1—O21	91.36 (11)	Cr1—OW1—H1B	135 (3)
O22—Cr1—O21	83.09 (11)	Cr1—OW1—H1A	117 (4)
O12—Cr1—O21	94.19 (11)	H1B—OW1—H1A	103 (4)
O11—Cr1—OW1	92.26 (11)	Cr1—OW2—H2A	123 (4)
O22—Cr1—OW1	89.69 (11)	Cr1—OW2—H2B	106 (4)
O12—Cr1—OW1	93.28 (11)	H2A—OW2—H2B	107 (3)
O21—Cr1—OW1	172.08 (11)	C2—N1—C3	122.5 (3)
O11—Cr1—OW2	171.04 (11)	C2—N1—H1	118.8
O22—Cr1—OW2	95.79 (11)	C3—N1—H1	118.8
O12—Cr1—OW2	89.07 (11)	N1—C2—C5	120.1 (4)
O21—Cr1—OW2	87.33 (11)	N1—C2—H2	120.0
OW1—Cr1—OW2	90.17 (12)	C5—C2—H2	120.0
O14—C11—O11	124.7 (3)	N1—C3—C4	119.4 (3)
O14—C11—C12	120.6 (3)	N1—C3—H3	120.3
O11—C11—C12	114.8 (3)	C4—C3—H3	120.3
O13—C12—O12	126.4 (3)	C3—C4—C6	119.4 (4)
O13—C12—C11	121.2 (3)	C3—C4—H4	120.3
O12—C12—C11	112.4 (3)	C6—C4—H4	120.3
O23—C21—O21	126.0 (3)	C2—C5—C6	119.2 (4)
O23—C21—C22	119.1 (3)	C2—C5—H5	120.4
O21—C21—C22	114.9 (3)	C6—C5—H5	120.4
O24—C22—O22	126.3 (3)	C5—C6—C4	119.4 (4)
O24—C22—C21	120.1 (3)	C5—C6—H6	120.3
O22—C22—C21	113.6 (3)	C4—C6—H6	120.3
C11—O11—Cr1	114.8 (2)		

### Hydrogen-bond geometry (Å, º)

**Table:**

*D*—H···*A*	*D*—H	H···*A*	*D*···*A*	*D*—H···*A*
N1—H1···O24	0.86	2.10	2.783 (4)	136
N1—H1···O23	0.86	2.17	2.901 (4)	143
O*W*1—H1*B*···O12^i^	0.81 (2)	1.94 (2)	2.720 (4)	162 (5)
O*W*1—H1*A*···O23^ii^	0.86 (5)	1.84 (5)	2.680 (4)	168 (5)
O*W*2—H2*A*···O13^iii^	0.81 (2)	1.98 (3)	2.732 (4)	154 (4)
O*W*2—H2*B*···O14^iv^	0.82 (2)	1.83 (2)	2.639 (4)	173 (5)

Symmetry codes: (i) *x*, −*y*+3/2, *z*+1/2; (ii) *x*, *y*, *z*+1; (iii) *x*+1, −*y*+3/2, *z*+1/2; (iv) *x*+1, *y*, *z*.
